# Improvement of Tryptophan Analysis by Liquid Chromatography-Single Quadrupole Mass Spectrometry Through the Evaluation of Multiple Parameters

**DOI:** 10.3389/fchem.2019.00797

**Published:** 2019-11-19

**Authors:** Rasmus la Cour, Henning Jørgensen, Jan K. Schjoerring

**Affiliations:** Department of Plant and Environmental Sciences, Faculty of Science, University of Copenhagen, Frederiksberg, Denmark

**Keywords:** high-throughput, HPLC, LC-MS, amino acid profile, protein

## Abstract

Tryptophan is a key component in many biological processes and an essential amino acid in food and feed materials. Analysis of the tryptophan content in proteins or protein-containing matrices has always been a challenge. We show here that the preparation of samples prior to tryptophan analysis can be significantly simplified, and the time consumption reduced, by using ascorbic acid as antioxidant to eliminate the problem of tryptophan degradation during alkaline hydrolysis. Combined with separation by HPLC and detection by Single Quadrupole Mass Spectrometry, this allows the analytical run time to be reduced to 10 min. The alkaline hydrolysate obtained in the method presented here may be combined with the oxidized hydrolysate obtained when sulfur-containing amino acids are to be measured, thus essentially providing two analyses for the time of one.

## Introduction

Many biological processes involve the essential amino acid tryptophan (Trp) or one of its metabolites. The Trp content of food and feed is therefore a very important factor for the growth (Liu et al., 2017) and general health and function of animals as well as humans (Friedman, 2018). Despite this fact, measurement of Trp has often been omitted in many studies of food and feed materials, because the Trp analysis has generally been tedious and only contributed with one additional amino acid to the full amino acid profile (Rozan et al., 2001; Bals et al., 2007; Dahl-Lassen et al., 2018; Gorissen et al., 2018). Decreasing the time consumption associated with the Trp analysis, as well as simplifying the protocol for the preparation of samples, may therefore encourage a wider use of Trp analysis, which in turn will lead to a better understanding of the nutritional quality of protein-containing products.

Recent advances in high-throughput analysis of the amino acid composition of proteins have led to a substantial reduction of the chromatographic run time compared to classical techniques (Dahl-Lassen et al., 2018). In combination with relatively cheap single quadrupole mass spectrometers, a significant simplification of amino acid analysis has been provided (Dahl-Lassen et al., 2018) compared to using more advanced mass spectrometers (Sakamoto et al., 2017; Han et al., 2018; Peris-Díaz et al., 2018; Tasakis and Touraki, 2018; Wang et al., 2018).

A major challenge of Trp analysis is that the acidic condition used to hydrolyze protein for normal amino acid analysis causes substantial or even complete oxidative degradation of Trp, which means that Trp cannot be quantified (Mason et al., 1979; Bech-Andersen, 1991). An alkaline hydrolysis has typically been implemented to partially solve this problem. While most methods use sodium hydroxide for hydrolysis (Bech-Andersen, 1991; Van Wickern et al., 1997; Yust et al., 2004; Zhang et al., 2009), other alkaline compounds, such as lithium hydroxide and barium hydroxide, have also been applied (Hugli and Moore, 1972; Lucas and Sotelo, 1980). Barium hydroxide is currently being used in commercial laboratories according to the ISO-standard for protein hydrolysis (ISO 13904:2005, 2005). Common for all these methods is that they need a laborious step to purge the sample in order to remove oxygen. This can be very time consuming and, depending on the nature of the sample, very difficult.

Oxidative degradation of Trp may also be prevented by addition of an antioxidant. Lactose and partially hydrolyzed starch have been tested and found to improve Trp yields (Hugli and Moore, 1972; Bech-Andersen, 1991), but are not considered antioxidants in a classical sense. Ascorbic acid is a naturally abundant and inexpensive antioxidant, making it suitable for routine analysis. It has been shown that ascorbic acid has a pro-oxidative effect on Trp in small amounts, but an antioxidative effect in larger amounts (Steinhart et al., 1993). Previously published methods, which have applied ascorbic acid as an antioxidant, have still included laborious steps such as flame sealing of reaction vessels in order to prevent oxygen from entering the hydrolysis mixture (Wu and Tanoue, 2001; Allenspach et al., 2018).

The purpose of the present study was to develop a method for high-throughput analysis of Trp in food, feed, and other protein-containing materials. In order to simplify the workflow, we especially tested the hypothesis that Trp analysis can be performed without removal of oxygen. We show that addition of ascorbic acid eliminates the need for tedious steps to prevent oxidation of Trp and that further sample preparation work-up after hydrolysis can be avoided by using lithium hydroxide. Pre-column derivatization of Trp is optional depending on sensitivity needs, but has the advantage that oxidized cysteine and methionine may be measured together with Trp, eliminating the need for one extra analysis on the HPLC system when determining a complete amino acid profile.

## Materials and Methods

### Materials

Analytical grade AccQ-Tag kit (containing acetonitrile, borate buffer, and 6-aminoquinolyl-N-hydrosysuccinimidyl carbamate reagent) was obtained from Waters (Millford, MA, USA). LC-MS grade acetonitrile, formic acid, lithium hydroxide, ascorbic acid, L-tryptophan, and cell free 13C-15N-labeled amino acid mixture were obtained from Sigma-Aldrich (St. Louis, MO, USA). Hydrochloric acid was obtained from Merck (Darmstadt, Germany). Milli-Q water (Millipore, Billerica, MA) was used for preparation of all buffers and reagents.

The matrices used in this study were whole shoots, juice, and pulp after extraction of red clover (*Trifolium pratense*), rye grass (*Lolium perenne*), and alfalfa (*Medicago sativa*), plant species representing potential biomasses for biorefineries. These materials were supplemented by shoots of stiff brome (*Brachypodium distachyon*), which is a model species in plant biology (Głazowska et al., 2018). In addition, seeds of triticale (×*Triticosecale*), leaves of spinach (*Spinacia oleracea*), a cheese from the local supermarket and dried dog food pellets were analyzed, representing food and feed materials. A certified reference material, NIST-1849a infant formula reference material, commercially available from National Institute of Standards and Technology (NIST) was also included.

### Preparation of Standard and Internal Standard Solutions

Tryptophan was weighed out and water added to a final stock concentration of 0.5 mM and frozen for storage (−20°C) in aliquots of 35 μL. From this stock, a calibration curve was prepared as described in the derivatization section.

The internal standard solution used was a complete mixture of 13C-15N labeled amino acids with a Trp concentration of 20 mM. The mixture was diluted to 0.5 mM and split into smaller aliquots for storage in the freezer (−20°C). This diluted mixture was ready to use.

### Sample Preparation

All plant materials and the dog food sample were freeze dried and ground by adding 3–5 steel spheres to the samples and shake them on a paint shaker until powdery (10–15 min) prior to analysis. The reference material was analyzed in the received condition.

### Hydrolysis During Optimization

Approximately 20 mg of sample was weighed into a 10 mL head space vial with crimp cap. The antioxidant was added to the sample prior to addition of the base. Lithium hydroxide (4 M), sodium hydroxide (2.5 or 4.2 M) or solid barium hydroxide octahydrate together with water (2.5 M) were added in a volume of 3 mL to the sample and the vials were sealed. A conventional oven preheated to 110°C was used for hydrolysis. Samples were left for the specified time. After hydrolysis was completed, the vials were taken out of the oven and cooled to handling temperature. Once cooled, the samples were neutralized using hydrochloric acid (6 M) and mixed thoroughly. In the case of hydrolysis with barium hydroxide, the neutralization was done using 0.5 mL of phosphoric acid (85%) and 1 mL hydrochloric acid (6 M). An aliquot of the samples was filtered through a syringe filter (0.45 μm) and derivatized as described below.

### Final Hydrolysis Protocol

Approximately 20 mg of sample was weighed into a 10 mL head space vial with crimp cap. Then 3.0 mL of a freshly prepared solution of 95 mM ascorbic acid in 4 M lithium hydroxide was added to the sample and the vial was sealed. The sealed vials were placed in a preheated oven (110°C) and left there for 16 h. After hydrolysis was completed, the vials were taken out of the oven and cooled to handling temperature. Once cooled, 2 mL hydrochloric acid (6 M) was added, and the samples were mixed thoroughly. An aliquot of the sample was filtered through a syringe filter (0.45 μm) and derivatized as described below.

### Derivatization With AccQ-Tag and Chromatography

Derivatization with the AccQ-Tag kit and chromatography were performed according to Dahl-Lassen et al. (2018) with modifications to the detection method. The samples were derivatized with aminoquinoline reagent (AQC) by mixing 5 μL of the sample with 33.5 μL of borate buffer, 1.5 μL of internal standard mixture (0.5 mM), and 10 μL of dissolved AQC reagent. The standard curve was prepared by mixing 8, 6, 5, 4, 3, 2, 1, 0.4, 0.2, and 0.1 μL, respectively with 3 μL of internal standard mixture and adding borate buffer to a volume of 80 μL. Then 20 μL of dissolved AQC reagent was added. Both standards and samples were mixed thoroughly immediately after addition of the AQC reagent. The tubes were then heated at 55°C for 10 min.

Sample analysis was performed on a Waters UPLC system with a Binary solvent manager and a sample manager. The detection of the amino acids was performed on a Waters QDa single quadrupole mass spectrometer in positive mode. The settings of the mass spectrometer was optimized to a cone voltage of 14 V and a mass of 375.2 m/z for unlabeled Trp and 388.2 m/z for the labeled internal standard. The mass spectrometer allowed only for unit resolution meaning that compounds within ±1 m/z of the target mass was detected.

Separation was carried out on a Cortecs UPLC C18 (1.6 μm particle size, 2.1 × 150 mm) column with a VanGuard Cortecs UPLC C18 (1.6 μm particle size, 2.1 × 5 mm) guard column. The column temperature was steady at 55°C and the injection volume was 1 μL. Gradient elution was used with 0.5% formic acid in water as eluent A and in acetonitrile as eluent B. The flow rate was constant at 0.500 mL min^−1^. The following gradient was used (expressed as solvent B): Initial conditions: 0.0% B, 0.0–0.54 min: 0.1% B, 0.54–4.00 min: 6.0% B, 4.00–4.50 min: 13.0% B, 4.50–7.50 min: 16.0% B, 7.50–8.04 min: 59.6% B, 8.04–8.05 min: 90.0% B, 8.05–8.64 min: 90.0% B, 8.64–8.73 min: 0.0% B, 8.73–10.00 min: 0.0% B.

### Calculation of Recovery

For calculation of recovery, nine replicates were measured over 3 days. All recoveries, except for the NIST reference material, were based on a comparison with results obtained by analyzing the same material in a commercial laboratory. The commercial laboratory is accredited by the Danish accreditation authorities and are performing the analysis in accordance with the generally accepted ISO 13904:2005 standard for analysis of Trp. The recoveries shown for the NIST reference material were compared to the certified values.

### Data Analysis

Data analysis was performed in Excel. Comparisons of sets of samples were analyzed using a student's *t*-test.

## Results and Discussion

### Specificity

**Figure 1:**
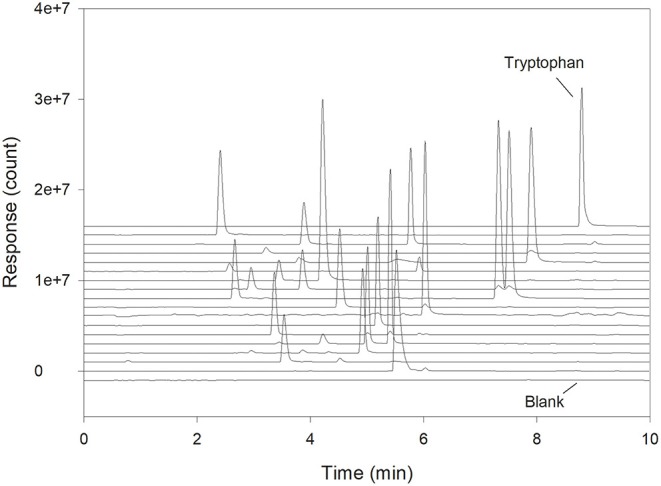
Chromatogram showing a full amino acid standard divided into mass traces. Each trace shows detection at a specific mass with Trp at the top and a blank sample at the bottom.

The specificity of the separation and detection method was tested by injection of a solution of Trp. A full amino acid standard was used to show that none of the other amino acids interfered with the measurements (Figure 1). This is important, as all amino acids will normally be present in the sample when analyzing Trp in a protein hydrolysate. Because Trp in this case was detected with a mass spectrometer (QDa detector) and not by fluorescence, molecules of other masses with the same retention time at ~9 min could not interfere. It is therefore very unlikely that any interferences would be present, as was also confirmed (Figure 1). Sample blanks treated as a normal sample and going through all the analytical preparations verified that no compounds were contaminating or co-eluting with Trp (blank sample trace, Figure 1). With the given analytical setup, the total analysis time was 11.5 min from injection to injection, but due to the specificity provided by the use of the QDa detector, an even shorter run time is possible.

### Calibration and Sensitivity

The influence of the cone voltage on the performance of the QDa detector was evaluated and the optimal value was found to be 14 V. The effect of formic acid concentration (0.1–1%) in the mobile phases was also tested. The best detector response (signal-to-noise ratio) was obtained using 0.5% formic acid (data not shown).

**Figure 2:**
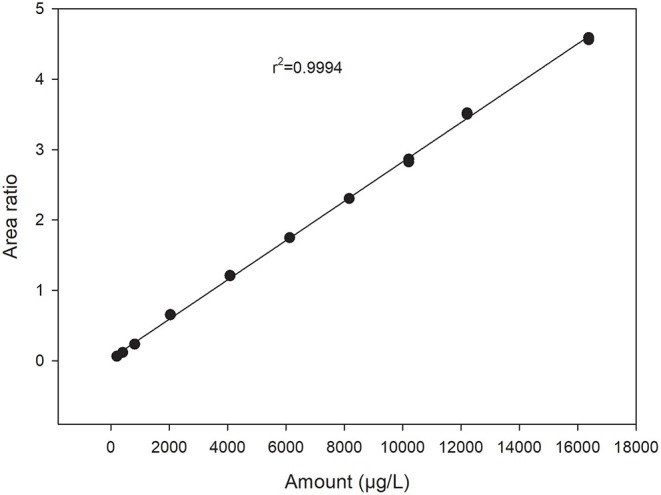
Calibration curve for tryptophan showing peak area relative to the internal standard peak area plotted against amount in μg/L. Two replicates analyzed at each standard level.

With the chosen setup, the working range was between 0.5 and 40 μM for Trp. This range was suitable for analyzing most materials without the need for diluting or concentrating the sample, thus decreasing the analytical sample preparation time. Within this working range, calibration curves for Trp showed a linear response (*r*^2^> 0.99) when a stable isotope labeled internal standard was included (Figure 2). Limit of Quantitation (LOQ) was calculated as 10 times signal-to-noise ratio and determined to be 0.06 μM. Limit of Detection (LOD) was calculated as three times signal-to-noise ratio and determined to be 0.02 μM. This was comparable to other methods described in literature with mass spectrometry detection (Liming et al., 2009; Gaudin et al., 2014; Tang et al., 2014).

On the QDa detector, Trp can be analyzed in the underivatized form. This will shorten the time of analytical preparation considerably, but the sensitivity will be lowered by approximately a factor of 10, giving a comparable sensitivity to other methods based on fluorescent or UV detection (Meussen et al., 2014; Tasakis and Touraki, 2018). Therefore, it was decided to continue with the derivatization step. Furthermore, the derivatization step was necessary for detection of the other amino acids in a combined analysis as described below.

### Evaluation of Optimal Hydrolysis Conditions

**Figure 3:**
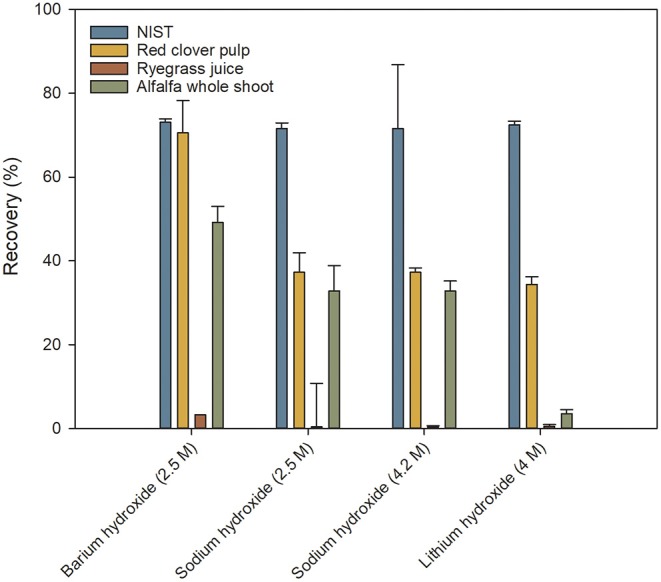
Recoveries of tryptophan following sample hydrolysis with different bases. Values are expressed relative to values obtained from commercial laboratories. Values are means ± S.D. (*n* = 9).

The efficiency of protein hydrolysis and recovery of Trp is a critical aspect. Several different setups of the hydrolysis were tested based on previously published methods. Different bases were tested for the alkaline hydrolysis (Figure 3), and the time of hydrolysis was varied (data not shown). Using a hydrolysis time between 8 and 22 h revealed no significant difference in the performance of the analysis. The result for the 4 h hydrolysis time was significantly lower than those for the longer times. This demonstrated a good robustness of the hydrolysis with regard to hydrolysis time and a standard time of 16 h was chosen.

From the results in Figure 3 it was clear that neither of the tested bases were able by themselves to yield the desired recovery of Trp after the hydrolysis. This was attributed to Trp being susceptible to degradation by oxidation. Therefore, antioxidants were added to the hydrolysis mixture. Samples hydrolyzed with sodium hydroxide were prone to turning into a gel when neutralized with hydrochloric acid. This made the subsequent filtering process exceedingly difficult. Sodium hydroxide was therefore discarded in the following optimization. Barium hydroxide showed the best results in the initial tests and is also the base used in the ISO certified method for Trp analysis (ISO 13904:2005, 2005). However, due to precipitation, barium was incompatible with the borate buffer used in the derivatization step. A further disadvantage of barium hydroxide is that it has to be added as a solid compound which, in a high-throughput setup, is much less desirable than dispensing a liquid. Barium hydroxide was therefore also discarded and lithium hydroxide was chosen for further optimization of the hydrolysis.

**Figure 4:**
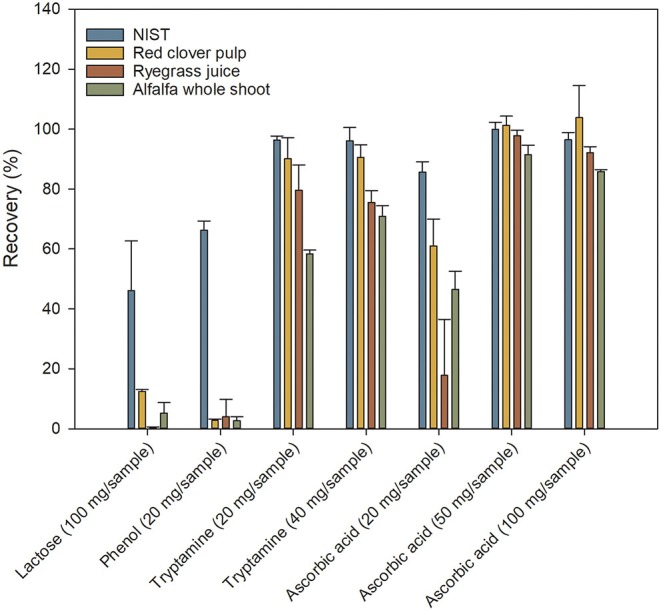
Efficiency of different antioxidants on tryptophan recovery. Recoveries are shown for four different antioxidants added in increasing concentrations. Values are expressed relative to values obtained from a certified commercial laboratory. Values are means ± S.D. (*n* = 9).

Lactose and phenol proved ineffective as antioxidants or stabilizers of Trp (Figure 4), whereas tryptamine and ascorbic acid had a strong positive influence. Dose-response of both tryptamine and ascorbic acid was investigated, but due to the low solubility of tryptamine in lithium hydroxide, increasing the concentration to 40 mg/sample, corresponding to 13.3 mg/ml in the hydrolysis solution, resulted in insoluble tryptamine even after completed hydrolysis. Therefore, it was only possible to study the effect of ascorbic acid, where an increase in concentration from 20 to 50 mg per sample significantly improved the Trp recovery. However, a further increase from 50 to 100 mg ascorbic acid per sample did not result in significantly improved Trp recovery (Figure 4). The recoveries obtained using 50 mg ascorbic acid per sample as antioxidant were generally good, ranging from 92 to 101%. The level of added ascorbic acid was higher than previously reported (Wu and Tanoue, 2001), potentially accounting for the increase in recovery even without having to remove oxygen from the sample/head space. The improvement in recovery due to addition of tryptamine or ascorbic acid as antioxidants was much higher for ryegrass juice and alfalfa whole shoot samples than for the red clover pulp (compare Figures 3, 4). The reason for this may be that the juice and whole plant samples contained more highly soluble compounds that acted as catalysts of the oxidative degeneration, and which had been removed from the pulp during the extraction process.

### Accuracy

Using the optimized hydrolysis described above, a set of nine different plant samples were tested for Trp recovery by comparing the obtained values with the values received from a certified commercial laboratory. Each sample was analyzed a total of 9 times over 3 days. The recoveries ranged from 90 to 98% of the values obtained by the commercial laboratory (Table 1), which is considered acceptable because it is within the uncertainty of the method. Furthermore, a certified reference material (NIST-1849a), used by commercial laboratories as the primary source for validation of accuracy, was analyzed with a recovery of 100%, strongly supporting the validity of the method. The average recovery for all the matrices was 95.5% (Table 1).

**Table 1:** Recoveries and relative standard deviation of Trp in different sample matrices.

**Sample**	**Recovery (%)**	**Relative standard deviation (%)**
Red clover juice	97.4	8.5
Red clover pulp	97.5	7.1
Red clover shoot	93.9	9.7
Ryegrass juice	96.5	7.2
Ryegrass pulp	95.0	7.7
Ryegrass shoot	89.9	8.5
Alfalfa juice	96.5	9.5
Alfalfa pulp	97.2	7.5
Alfalfa shoot	91.5	10.3
Spinach leaves	N.D.	7.7
Triticale seeds	N.D.	8.3
*Brachypodium* shoot	N.D.	8.0
Pet food	N.D.	7.9
NIST-1849a	100	7.5
Cheese	N.D.	8.5
Average	95.5	8.2

*N.D., Not determined. These samples were not part of the sample set sent to the certified commercial laboratory*.

*Relative standard deviation based on nine repetitions over 3 days, including all parts of the analysis*.

*Recoveries are relative to values from a certified commercial laboratory, except for NIST-standard recovery, which is relative to the certified value*.

### Reproducibility

The reproducibility was tested on 15 different sample types. Each sample type was analyzed in triplicates on three different days. The average relative standard deviation of the 15 sample types was 8.3%, ranging from 7.1 to 10.3% (Table 1). This standard deviation was slightly larger than other examples seen in the literature (Wu and Tanoue, 2001; Armstrong et al., 2007; Zhang et al., 2009), but still comparable. By analyzing 10 injections from the same vial, the contribution to the uncertainty coming from the HPLC system and MS-detection was determined to be 3.3%. By derivatizing and analyzing the same sample five times, the contribution to the uncertainty from the derivatization step was found to be 2.8%. The remaining uncertainty was therefore related to the hydrolysis step and the day-to-day variation. Only a few methods presented in the literature consider the hydrolysis step (Allenspach et al., 2018), thereby neglecting a major source of variation.

### Combined Analysis of Cysteine, Methionine, and Trp

Analysis of the sulfur-containing amino acids methionine and cysteine requires that they are protected from degradation during standard acid hydrolysis. This is achieved by a pre-hydrolysis step in which performic acid is added to the sample, resulting in oxidation of the two amino acids to methionine sulfone and cysteic acid, respectively. During the oxidative hydrolysis, Trp is degraded, implying that no Trp is present in the hydrolysate. In the alkaline hydrolysis carried out in connection with Trp analysis, ascorbic acid as an antioxidant is preventing the formation of methionine sulfone and cysteic acid. The two hydrolysates from the oxidative and the alkaline hydrolysis might therefore be combined before derivatization, which would allow Trp, cysteine (cysteic acid), and methionine (methionine sulfone) to be quantified simultaneously in one analytical run. We tested this on a sample set of protein concentrates from plant material along with different whole plant materials and the certified reference material (NIST-1849a).

For Trp and methionine, there was no significant difference in quantified amount between the normal single and the combined analyses (*p* = 0.27 and *p* = 0.26, respectively). The combined analysis showed a slight tendency (*p* = 0.024) toward overestimating the content of cysteine. The average overestimation was 6.4%, which was within the range of the uncertainty of cysteine analysis in the method developed by Dahl-Lassen et al. (2018). The difference was mainly attributed to certain sample types as shown by the fact that the cysteine content of the *Brachypodium* samples and alfalfa samples were overestimated by 17 and 16% respectively, while the difference observed for the other samples, including the reference material, was not significant. The overestimation of cysteine may be caused by cysteine acting as an antioxidant, as has been demonstrated previously (Allenspach et al., 2018). Partial oxidation of cysteine to cysteic acid may be overcome by addition of extra ascorbic acid, however this has been demonstrated to have an adverse effect on the recovery of Trp in some sample types (Allenspach et al., 2018).

The combined method is accordingly considered suitable for most sample types, but it is strongly recommended that each sample type is tested before the combined method is applied.

## Conclusion

The analytical protocol presented here makes the analysis of Trp more applicable for high throughput setups. This was achieved by using ascorbic acid as an antioxidant during the hydrolysis of proteins, thereby eliminating the need for the labor-intensive steps of removing oxygen from the sample and the atmosphere in the hydrolysis vial. The option of pre-column derivatization allows the user a choice of improved sensitivity or minimal active analytical preparation time. Combining the alkaline hydrolysate for Trp determination with the oxidized hydrolysate for methionine and cysteine determination prior to analysis by Single Quadrupole Mass Spectrometry, will further decrease the analytical preparation time, as well as the analytical run time, essentially resulting in a “free” analysis in terms of both time and material costs.

## Data Availability Statement

The datasets generated for this study are available on request to the corresponding author.

## Author Contributions

RC developed and performed the experimental work. All authors contributed to the design of the work, the writing of the manuscript, and read and approved the final manuscript.

### Conflict of Interest

The authors declare that the research was conducted in the absence of any commercial or financial relationships that could be construed as a potential conflict of interest.
